# A Retrospective Review of a Single-Center Experience with Posterolateral Fundoplication During Esophagogastrostomy After Proximal Gastrectomy

**DOI:** 10.1007/s11605-021-05052-8

**Published:** 2021-07-08

**Authors:** Masaki Aizawa, Hiroshi Yabusaki, Koji Nakada, Atsushi Matsuki, Takeo Bamba, Satoru Nakagawa

**Affiliations:** 1 Department of Digestive surgery, Niigata Cancer Center Hospital, 2-15-3, Kawagishicho, Niigata, 951-8566 Japan; 2 Department of Laboratory Medicine, The Jikei University School of Medicine, 3-25-8, Nishishimbashi, Minato-ku, Tokyo 105-8461 Japan

**Keywords:** Proximal gastrectomy, Esophagogastrostomy, Post-gastrectomy syndrome, Quality of life, Reflux esophagitis, Fundoplication

## Introduction

For patients with proximal gastric cancer (PGC), proximal gastrectomy (PG) has been proposed as a surgical option with the expectation of ameliorating post-gastrectomy syndrome. Although esophagogastrostomy with a large remnant stomach is a simple reconstruction method that allows gastric functions and capacity to accommodate food to be preserved, patients undergoing this procedure often develop severe reflux esophagitis.^1^, ^2^ At present, esophagogastrostomy with an additional antireflux procedure is considered as the most promising reconstruction technique.^3^–^5^ Herein, we report our experience with the addition of the posterolateral fundoplication (PLF) technique to the standard esophagogastric anastomosis.

## Methods

**Fig. 1:**
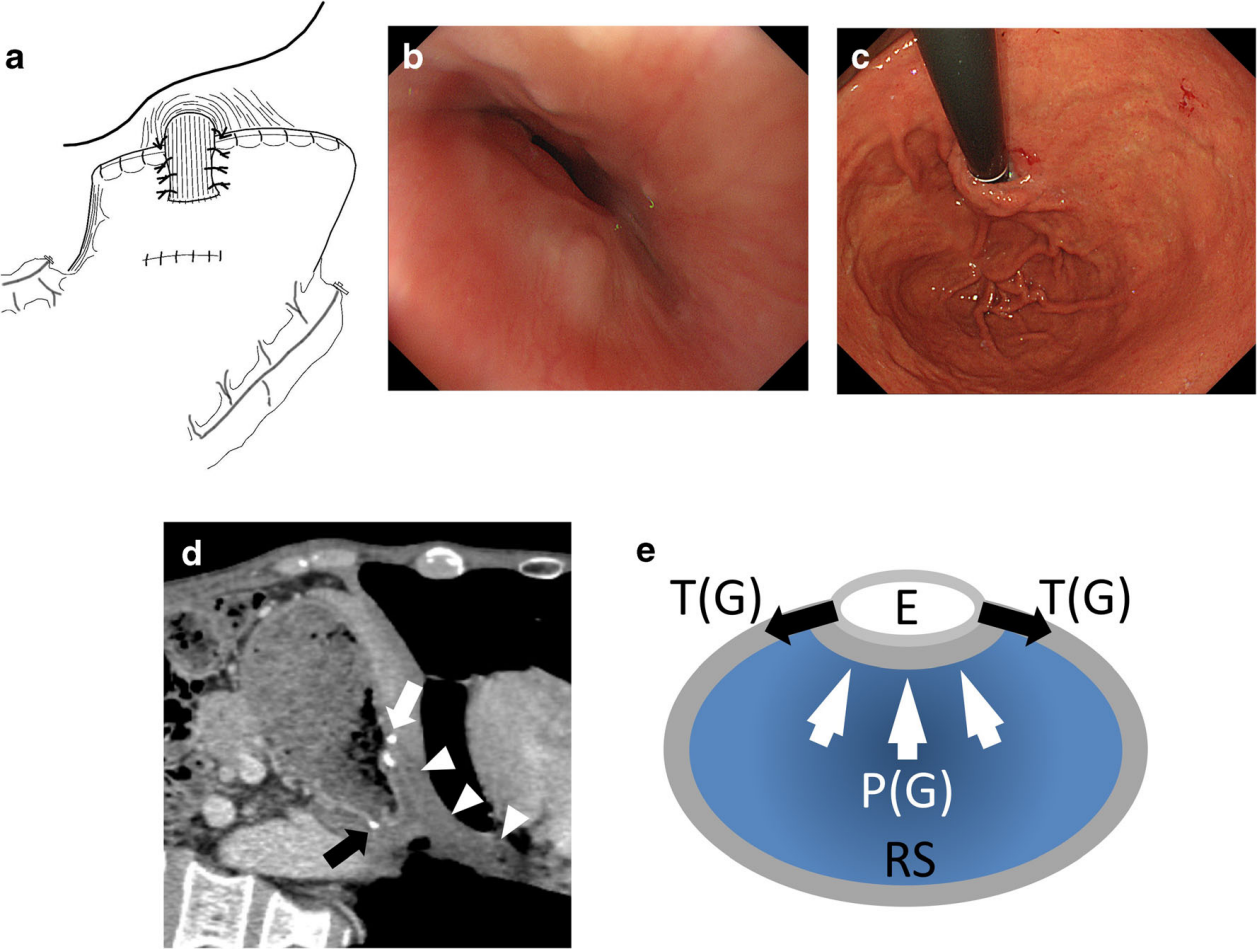
Schema of posterolateral fundoplication after esophagogastrostomy (**a**), endoscopic view the of anastomosis from the esophagus (**b**) and stomach (**c**) at the 3-years follow-up after surgery, longitudinal-section CT image at the 3-year follow-up after surgery (**d**), white arrow: anastomosis, black arrow: gastric stump, white arrowhead: esophagus, Coronal-section image of the abdominal esophagus overlapping the remnant stomach and mechanical actions (**e**), E: esophagus; RS: remnant stomach; P(G): internal pressure of the remnant stomach; T(G): traction by stretching of the stomach

The schema of PLF is shown in Fig. 1a. After PG with D1+ regional lymphadenectomy, esophagogastrostomy using a mechanical circular stapler was performed at the anterior wall of the remnant stomach, the center of which was up to a length of 40 mm from the cut end of the stomach. Then, the cut end of the stomach was fixed to both the top posterior end of the freed esophageal wall and the diaphragm. Finally, the posterior half-circumference of the esophagus was wrapped with the anterior gastric wall by placing stay sutures.

After obtaining approval from the ethics board for this study, the data of 28 patients who underwent PLF between July 2016 and December 2019 was reviewed.

## Results

The clinicopathological features and postoperative findings are summarized in Table 1. The surgery was performed by the laparoscopic approach in 23 patients. Seven patients developed benign membranous stricture several months after surgery, all of the seven patients were successfully treated by endoscopic mechanical dilatation. The PLF was maintained during over years (Fig. 1b–d). None of the patients developed either endoscopic or clinical evidence of reflux esophagitis.

**Table 1:** Patient characteristics, tumor-related factors and surgical outcome

Characteristics		Total N = 28
Age (year)	Mean (SD)	69.1 (8.81)
Gender, N (%)	Male Female	26 (92.9) 2 (7.1)
Disease, N (%)	Adenocarcinoma NEC GIST	27 (96.4) 1 (3.6) 0 (0)
Body mass index	Mean (SD)	23.5 (3.26)
Pathological T stage, N (%)	1a 1b 2 3	5 (17.9) 19 (67.8) 3 (10.7) 1 (3.6)
Pathological N stage, N (%)	0 1 2	25 (89.2) 2 (7.2) 1 (3.6)
Pathological stage, N (%)	IA IB IIA IIB	23 (82.1) 3 (10.7) 0 (0.0) 2 (7.2)
Surgical approach, N (%)	Open method Laparoscopic	5 (17.9) 23 (82.1)
Operation time (min)	Median (range)	296 (215–406)
Blood loss during surgery	Median (range)	25 (3–800)
Postoperative morbidity (≥Clavien-Dindo grade III), N (%)	None Anastomotic stenosis	21 (75.0) 7 (25.0)
Body weight loss a year after surgery, %	Mean (SD)	−10.7 (6.6)
Period of hospital stay after surgery (date)	Median (range)	9 (7–22)
Endoscopic evidence of esophagitis, N (%)	Any grade of LA classification	0 (0)
Reflux symptom after surgery	- +	28 (100) 0 (0.0)
Recurrence after surgery	- +	28 (100) 0 (0.0)
Observation period, month	Median (range)	22.5 (12.0–43.4)

*NEC*, Neuroendocrine carcinoma; *GIST*, Gastrointestinal stromal tumor; *LA*, Los Angeles

## Discussion

The PLF procedure is simple. The proposed physiology underlying the antireflux effect of PLF is shown in Fig. 1e. The 180° wrapping of the esophagus with the anterior gastric wall forms the internal valve. Furthermore, the stretch of the gastric wall pulls the side wall of the esophagus on either side in the lateral direction. While the remnant stomach becomes filled with contents, the lumen of the esophagus closes, and does not open until the remnant stomach becomes empty.

Though the safety of PLF was acceptable, development of anastomotic stenosis was the lone worrisome event. Stenosis developing after mechanical esophagogastrostomy has been reported to be not life-threatening and as being treatable by mechanical dilatation.^6^

Patients in whom the entire abdominal esophagus and more than half of the stomach can be preserved are a suitable indication of PLF. In regard to the oncological safety, the non-inferiority of PG to total gastrectomy remains to be established. Hence, in Japan, the indication of PG for patients with PGC is generally limited to patients with clinical stage T1N0 disease.

There were limitations of the present study. First, this was a retrospective study with a small sample size. Second, supportive evidence to validate the rationale for PLF is still lacking. Third, a comparison of different antireflux procedures is needed to establish the optimal antireflux procedure.

## Conclusion

Esophagogastrostomy with PLF is a promising reconstruction procedure after PG.

## References

[1] An JY, Youn HG, Choi MG, Noh JH, Sohn TS, Kim S (2008). The difficult choice between total and proximal gastrectomy in proximal early gastric cancer. Am J Surg.

[2] Ronellenfitsch U, Najmeh S, Andalib A, Perera RM, Rousseau MC, Mulder DS, Ferri LE (2015). Functional outcomes and quality of life after proximal gastrectomy with esophagogastrostomy using a narrow gastric conduit. Ann Surg Oncol.

[3] Inada T, Yoshida M, Ikeda M, Yumiba T, Matsumoto H, Takagane A, Kunisaki C, Fukushima R, Yabusaki H, Nakada K (2014). Evaluation of QOL after proximal gastrectomy using a newly developed assessment scale (PGSAS-45). World J Surg.

[4] Kuroda S, Nishizaki M, Kikuchi S, Noma K, Tanabe S, Kagawa S, Shirakawa Y, Fujiwara T (2016). Double-Flap Technique as an Antireflux Procedure in Esophagogastrostomy after Proximal Gastrectomy. J Am Coll Surg.

[5] Yamashita Y, Yamamoto A, Tamamori Y, Yoshii M, Nishiguchi Y (2016). Side overlap esophagogastrostomy to prevent reflux after proximal gastrectomy. Gastric Cancer.

[6] Yuan Y, Wang KN, Chen LQ (2015). Esophageal anastomosis. Dis Esophagus.

